# Determinants of patient adherence: a review of systematic reviews

**DOI:** 10.3389/fphar.2013.00091

**Published:** 2013-07-25

**Authors:** Przemyslaw Kardas, Pawel Lewek, Michal Matyjaszczyk

**Affiliations:** First Department of Family Medicine, Medical University of LodzLodz, Poland

**Keywords:** medication adherence, patient compliance, persistence, concordance, medication use, determinants of adherence

## Abstract

**Purpose:** A number of potential determinants of medication non-adherence have been described so far. However, the heterogenic quality of existing publications poses the need for the use of a rigorous methodology in building a list of such determinants. The purpose of this study was a systematic review of current research on determinants of patient adherence on the basis of a recently agreed European consensus taxonomy and terminology.

**Methods:** MEDLINE, EMBASE, CINAHL, Cochrane Library, IPA, and PsycINFO were systematically searched for systematic reviews published between 2000/01/01 and 2009/12/31 that provided determinants on non-adherence to medication. The searches were limited to reviews having adherence to medication prescribed by health professionals for outpatient as a major topic.

**Results:** Fifty-one reviews were included in this review, covering 19 different disease categories. In these reviews, exclusively assessing non-adherence to chronic therapies, 771 individual factor items were identified, of which most were determinants of implementation, and only 47—determinants of persistence with medication. Factors with an unambiguous effect on adherence were further grouped into 8 clusters of socio-economic-related factors, 6 of healthcare team- and system-related factors, 6 of condition-related factors, 6 of therapy-related factors, and 14 of patient-related factors. The lack of standardized definitions and use of poor measurement methods resulted in many inconsistencies.

**Conclusions:** This study provides clear evidence that medication non-adherence is affected by multiple determinants. Therefore, the prediction of non-adherence of individual patients is difficult, and suitable measurement and multifaceted interventions may be the most effective answer toward unsatisfactory adherence. The limited number of publications assessing determinants of persistence with medication, and lack of those providing determinants of adherence to short-term treatment identify areas for future research.

## Introduction

Enormous progress in the fields of both medicine, and pharmacology has taken place in the last century and led to a completely new paradigm of treatment. Contrary to the past, in which most treatments were only available in hospitals, effective remedies are available now in ambulatory settings. At the same time, the demographic changes that happen to both developed and developing countries, make chronic conditions even more prevalent. All this makes the most modern treatments dependent on patient self-management. Surprisingly often, evidence based treatments fail to succeed because of the human factor known for a few decades as *patient non-adherence*.

Currently, sound theoretical foundations for adherence-enhancing interventions are lacking (van Dulmen et al., 2007). Therefore, the development of interventions to enhance patient adherence to medication, and maintain long term persistence, requires at least an understanding of the determinants of patient non-adherence to prescribed therapies. This is especially important when the determinants are modifiable risk factors, which, once identified, can then be targeted for beneficial changes.

The published literature identifies hundreds of determinants of non-adherence. Unfortunately, serious drawbacks of the methodology used by numerous studies demand that this list be revised. In particular, many studies do not indicate the relative importance of the 3 identified components of patient adherence: initiation, implementation, and discontinuation. For example, the WHO recommends that determinants be classified in 5 dimensions (Sabate, 2003): socio-economic factors, healthcare team and system-related factors, condition-related factors, therapy-related factors, and patient-related factors, but provides little or no closure in respect to outcome, and in particular, to the stage of adherence process. Moreover, little information exists on the determinants of short-term adherence for acute diseases vs. long-term adherence for chronic diseases.

The objective of this study was to identify and classify the determinants of non-adherence to short-term and long-term treatments for different clinical sectors and population segments. In order to obtain this goal, a retrospective systematic review of the literature was performed, wherein we have adopted the method of reviewing reviews. In order to design a comprehensive, yet evidence-based list of determinants of patient adherence for use in both practical and clinical settings, as well as for theoretical purposes to inform adherence-enhancing interventions, a rigorous taxonomy and terminology of adherence was used, the basis of which was set recently in a form of a European consensus (Vrijens et al., 2012). According to this terminology, *adherence to medications* is defined as the process by which patients take their medications as prescribed. Adherence has three components: *initiation*, *implementation*, and *discontinuation*, of which *initiation* is defined as the moment at which the patient takes the first dose of a prescribed medication; the *implementation* of the dosing regimen, being the extent to which a patient's actual dosing corresponds to the prescribed dosing regimen from initiation until the last dose taken; and *discontinuation*, being the end of therapy, when the next dose to be taken is omitted and no more doses are taken thereafter (Vrijens et al., 2012).

This study is part of a larger project on patient medication adherence funded by the European Commission called the “ABC (Ascertaining Barriers for Compliance) Project” (http://www.abcproject.eu). The overall goal of the ABC project was to produce evidence-based policy recommendations for improving patient adherence and by so doing to promote safer, more effective and cost-effective medicines use in Europe.

## Methods

As the number of publications with the keyword “patient compliance” (text word), and “patient compliance” as MESH major term is so high, (close to 50,000 hits, and 16,000 in PubMed by 2009/12/31, respectively), only recent systematic reviews are included in this search; More precisely the inclusion criteria comprised systematic reviews in the English language, published between 2000/01/01 and 2009/12/31, having adherence to medication intended to be taken in outpatient settings prescribed by health professionals, as a major topic of publication, if determinants of adherence are provided.

MEDLINE (through PubMed), EMBASE, CINAHL, the Cochrane Library, International Pharmaceutical Abstracts (IPA), and PsycINFO were searched for relevant publications. In order to increase search coverage, a number of possible synonyms for *medication adherence* (i.e., *patient compliance, concordance, patient dropouts, treatment refusal*, and *directly observed therapy*), in combination with several synonyms of *determinants* were used for keywords. The detailed search strategies for all databases are provided in Appendix 1. For the other databases, the search strategies were adapted accordingly.

Papers were excluded for the following reasons: (1) Studies that primarily focused on adherence-enhancing interventions. (2) Studies that were not systematic reviews. (3) Studies that assessed adherence to non-medication intervention (e.g., vaccination). (4) Double citations. (5) Determinants of adherence to medication not provided. No paper was excluded on the grounds of quality.

Eligibility assessment of title and abstract was performed independently in an unblended standardized manner by two reviewers (PK, PL). If at least one reviewer coded a review as potentially eligible, the review was included for full-text review. The full texts of potentially eligible reviews were retrieved and reviewed by both reviewers. Disagreements were resolved by discussion and a final decision was reached between the two reviewers.

A structured data collection sheet was developed to extract data from each review. All available relevant data was extracted from the reviews; no additional information was sought from the authors. The following paragraphs describe which data was extracted.

### Determinants of adherence to medication

A range of determinants were extracted based on the source publications. These were further categorized according to their effect on adherence to medication using an adherence determinant matrix. Relevant dimensions included:

- *Treatment duration: long*- vs. *short-term* treatment;
- *Components of adherence to medication*: *implementation* of the dosing regimen (defined as the extent to which a patient's actual dosing corresponds to the prescribed dosing regimen) vs. *persistence* (defined as the length of time between initiation and the last dose which immediately precedes discontinuation) (Vrijens et al., 2012). Determinants were categorized under implementation unless original review wording clearly addressed persistence.
- *Dimensions of adherence:* these were *socio-economic factors, healthcare team- and system-related factors, condition-related factors, therapy-related factors, and patient-related factors*. In this was original WHO report description followed (Sabate, 2003), with a modification: demographic variables were included under patient-related, instead of socio-economic related factors.
- *Direction of effect*: determinants were classified according to their *positive, negative, neutral*, or *not defined* effect on adherence.

Other data extracted from the reviews included scope of the review (medical condition, class of drugs, etc.), studied population, and databases searched by the authors.

## Results

**Figure 1:**
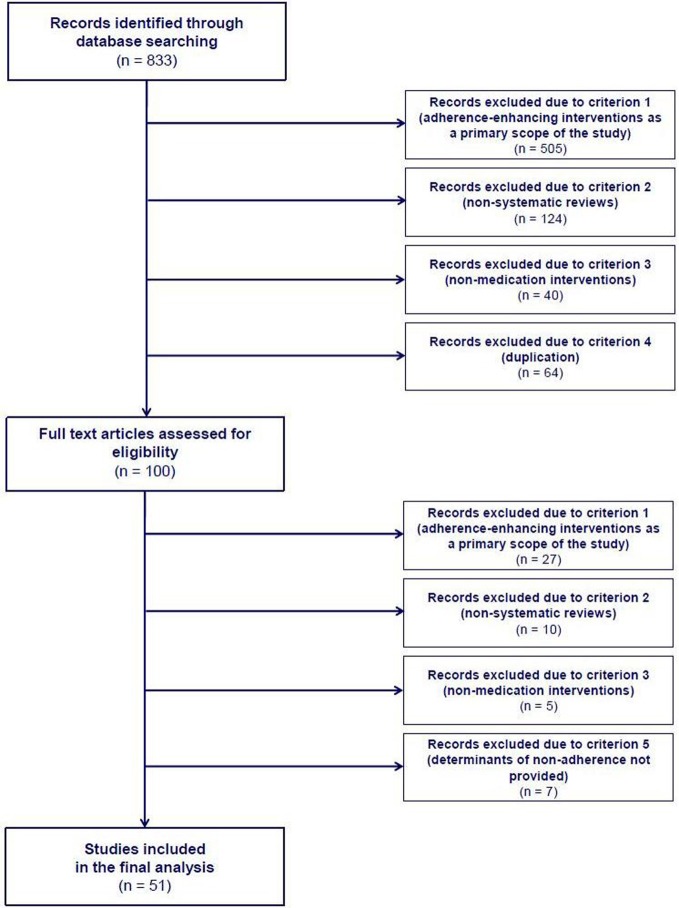
**Flow diagram of study selection process**.

In this systematic literature review, 51 systematic reviews were found to contain determinants of adherence to medication. An overview of the review process and reasons for exclusion at various steps within it are detailed in Figure 1. Individual study characteristics are listed in Appendix 2. Great variety was seen in both the start year of the literature searches performed within the source reviews, starting back from as early as 1948, or as late as 2000, as well as the period covered by the search, varying from 5 to over 50 years. Most of the studies accepted broad definitions of adherence, making no distinction between intentional, and unintentional non-adherence; only in 4 studies were clear operational definitions provided (Iskedjian et al., 2002; Lacro et al., 2002; Wetzels et al., 2004; Bao et al., 2009; Parienti et al., 2009). The majority of the studies were systematic reviews. However, 8 reviews (DiMatteo et al., 2000, 2007; Iskedjian et al., 2002; DiMatteo, 2004a,b; Gonzalez et al., 2008; Bao et al., 2009; Parienti et al., 2009) were also enriched with meta-analyses, and provided calculations of the effect on adherence of factors from several dimensions:

- *Socio-economic factors:* practical social support (OR 3.60, 95%CI 2.55 - 5.19), emotional support (OR 1.83, 95%CI 1.27, 2.66], unidimensional social support (OR 2.35, 95%CI 1.76–3.03], family cohesiveness (OR 3.03, 95%CI 1.99–4.52], being married (OR 1.27, 95%CI 1.12–1.43), as well as living with someone (for adults, OR 1.38, 95%CI 1.04–1.83) increased the odds of adherence, whereas family conflict decreased these odds (OR 2.35, 95%CI 1.08, 5.71) (DiMatteo, 2004a)
- *Condition-related factors:* patients rated poorer in health by their physicians were more adherent to treatment (OR 1.76, 95%CI 1.13 - 2.77) (DiMatteo et al., 2007)
- *Therapy-related factors:* the average adherence rate for QD dosing was significantly higher than for BID dosing in hypertension (92.7% vs. 87.1%) (Iskedjian et al., 2002) and antiretroviral therapy (+2.9%, 95%CI 1.0-4.8%) (Parienti et al., 2009), in hypertension adherence was also significantly higher for QD dosing vs. >QD dosing (91.4 vs. 83.2%, respectively) (Iskedjian et al., 2002). In methadone treatment, persistence was higher with higher daily methadone doses (> or =60 mg vs. <60 mg/day, OR: 1.74, 95%CI 1.43-2.11), as well as with flexible-dose strategies vs. fixed-dose strategies (OR: 1.72, 95%CI 1.41–2.11) (Bao et al., 2009)
- *Patient-related factors:* an extensive review found older age, female gender, higher income, and more education to have small yet positive effects on adherence (DiMatteo, 2004b). A belief that the medical condition in question was a threat because of its severity increased the odds of adherence (OR 2.45, 95%CI 1.91–3.16) (DiMatteo et al., 2007). Depression was significantly associated with non-adherence across various conditions (OR 3.03, 95%CI 1.96–4.89) (DiMatteo et al., 2000), and in particular, in diabetes (z 9.97, P 0.0001) (Gonzalez et al., 2008).

Within our selected reviews, the most common focus of the studies were miscellaneous diseases (10 reviews) (DiMatteo et al., 2000, 2007; Claxton et al., 2001; Vermeire et al., 2001; Connor et al., 2004; DiMatteo, 2004a,b; Vik et al., 2004; Chia et al., 2006; Kruk and Schwalbe, 2006), followed by HIV (8 reviews) (Fogarty et al., 2002; Mills et al., 2006; Malta et al., 2008; Vreeman et al., 2008; Lovejoy and Suhr, 2009; Parienti et al., 2009; Ramos, 2009; Reisner et al., 2009), and psychiatric conditions (8 reviews) (Oehl et al., 2000; Lacro et al., 2002; Pampallona et al., 2002; Nosé et al., 2003; Santarlasci and Messori, 2003; Charach and Gajaria, 2008; Julius et al., 2009; Lanouette et al., 2009) (Table 1). Disease categories were broad (19 different diseases); reviews exclusively reported patients with chronic diseases.

**Table 1:** **Fields covered by the selected reviews**.

**Field**	**No. of reviews**
Miscellaneous diseases	10
HIV	8
Psychiatric conditions	8
Diabetes	3
Hypertension	3
Cancer	2
End stage renal disease	2
Multiple sclerosis	2
Osteoporosis	2
Transplantations	2
Tuberculosis	2
Cystic fibrosis	1
Skin diseases	1
Glaucoma	1
Heart failure	1
Malaria	1
Opioid dependence	1
Non-malignant chronic pain	1

Close to half of the reviews (25 out of 51) did not specify the age group of patients covered by the review. Of the rest, most dealt with adults (11 reviews, Table 2).

**Table 2:** **Patient groups covered by the selected reviews**.

**Patient group**	**No**.
Not specified	25
Adults	11
Children + adults	8
Children	4
Elderly	2
Youth	1

### Determinants of adherence to medication

As many as 771 individual factor items associated with long-term treatment were extracted from the reviewed literature: Despite the broad range of the fields covered with these publications, no publication primarily focusing on short-term therapies was identified, nor were any individual determinants of patient adherence to short-term treatment. The vast majority of individual factor items were determinants of implementation, and only 47 were found to be determinants of persistence with medication. Only three reviews addressed the *initiation* component of adherence, although no corresponding determinants were provided (Vermeire et al., 2001; Vik et al., 2004; Costello et al., 2008).

For 64 individual factor items, no unambiguous information concerning their effect on adherence to medication could be found in the source publication. The remaining factors were grouped to form 400 individual determinants: 143 with a positive, 155 with negative, and 102 with neutral effect on adherence. In cases where the source publications provided two “mirror” versions of the same factor, e.g., *family support* and *lack of family support*,, these were recategorized as the factor with a negative effect on adherence, in this case, *lack of family support*. The determinants were further clustered according to the modified WHO 5 dimension of adherence (see Methods for details). The results are presented in Tables 3–7 as socio-economic-related factors (8 clusters), healthcare team- and system-related factors (6 clusters), condition-related factors (6 clusters), therapy-related factors (6 clusters), and patient-related factors (14 clusters).

**Table 3:** **Socio-economic factors affecting adherence**.

**Factors having**		
**Negative effect on adherence**	**Positive effect on adherence**	**Neutral effect on adherence**
**FAMILY SUPPORT**		
Lack of family support (Nosé et al., 2003; Munro et al., 2007; Costello et al., 2008) Irregular supervision by a family member (Munro et al., 2007^P^) Child selfresponsibility for taking medication (Kahana et al., 2008)	Family financial support (Munro et al., 2007; Lanouette et al., 2009) Family support in executing medication (Oehl et al., 2000; Munro et al., 2007; Lanouette et al., 2009)	Family emotional support (Weiner et al., 2008; Lanouette et al., 2009) Family involvement during hospitalization or follow-up (Lacro et al., 2002)
**FAMILY/CAREGIVERS FACTORS**		
Disorganized biologic families (Kahana et al., 2008; Karamanidou et al., 2008; Vreeman et al., 2008) Family in conflict (Oehl et al., 2000; DiMatteo, 2004a; Vreeman et al., 2008; Weiner et al., 2008) Responsibilities in the home (such as providing income and caring for children) (Munro et al., 2007) Low parental educational level (Vreeman et al., 2008) Family beliefs about the nature of the patient's illness (Julius et al., 2009) More people in household (in children) (DiMatteo, 2004a) Having several adults involved in pill supervision (Vreeman et al., 2008)	Two-parent families (Charach and Gajaria, 2008^P^) Family cohesiveness (DiMatteo, 2004a) Having an adult other than the biologic parent as primary caregiver (Reisner et al., 2009) Higher caregiver education level (Reisner et al., 2009) Responsibilities in the family (Munro et al., 2007) Parental belief that ADHD is a biological condition (Charach and Gajaria, 2008^P^) Mother's perception of the severity of disease (Hodari et al., 2006)	Knowledge of family members regarding disease (Lacro et al., 2002) Family member with mental illness (Lanouette et al., 2009) Number of people in the household (Vermeire et al., 2001) Parental marital status (Charach and Gajaria, 2008^P^)
**SOCIAL SUPPORT**		
Lack of social support (Oehl et al., 2000; Fogarty et al., 2002; DiMatteo, 2004a; Mills et al., 2006; Costello et al., 2008; Hirsch-Moverman et al., 2008; Karamanidou et al., 2008; Malta et al., 2008; Weiner et al., 2008; Julius et al., 2009; Schmid et al., 2009) Less acculturation (Lanouette et al., 2009) Low social functioning (Nosé et al., 2003) Low social rank of an illness (Oehl et al., 2000) Negative publicity regarding HAART or the medical establishment (Mills et al., 2006)	Emotional support (DiMatteo, 2004a) Good social adjustment (Pampallona et al., 2002; Nosé et al., 2003) Including significant others into therapeutic alliance (Oehl et al., 2000) Supervision of medication administration by others (Weiner et al., 2008; Julius et al., 2009) Patients' support to patients (Munro et al., 2007; Costello et al., 2008)	Social support (Reisner et al., 2009)
**SOCIAL STIGMA OF A DISEASE**		
Stigma of a disease at school, at workplace, among the family and friends (Munro et al., 2007; Vreeman et al., 2008; Reisner et al., 2009) Negative attitude in the patient's social surroundings toward psychiatric treatment (Oehl et al., 2000) Fear of disclosure and wanting to avoid taking medications in public places (Mills et al., 2006) Disclosure of the child's HIV status (Vreeman et al., 2008) Hiding the disease (TB) for fear that employers may discover it (Munro et al., 2007)	Openly disclosing HIV status to family and friends (Mills et al., 2006)
**COSTS OF DRUGS AND/OR TREATMENT**		
Cost of drugs (co-payment) (Vermeire et al., 2001; Gold et al., 2006^P^; Hodari et al., 2006; Lewiecki, 2007; Vreeman et al., 2008; Schmid et al., 2009) Costs of drugs and treatment (Munro et al., 2007^P^; Costello et al., 2008^P^)		
**PRESCRIPTION COVERAGE**		
Lack of, or inadequate medical/prescription coverage (Charach and Gajaria, 2008^P^; Costello et al., 2008^P^; Lanouette et al., 2009; Schmid et al., 2009) Fear of asking for money from employer to purchase drugs (in TB) (Munro et al., 2007)	Having health insurance (Lanouette et al., 2009)	
**SOCIOECONOMIC STATUS**		
Low income (Jindal et al., 2003; DiMatteo, 2004b; Munro et al., 2007; Schmid et al., 2009) Poverty (Munro et al., 2007^P^; Costello et al., 2008^P^; Vreeman et al., 2008) Lower socioeconomic status (DiMatteo, 2004b; Charach and Gajaria, 2008^P^; Lanouette et al., 2009) Financial constraints (Oehl et al., 2000; Mills et al., 2006) Wanting to remain sick to qualify for financial support (Munro et al., 2007)		Socioeconomic status (Vermeire et al., 2001; Munro et al., 2007; Charach and Gajaria, 2008^P^; Karamanidou et al., 2008; Weiner et al., 2008; Ruddy et al., 2009) Financial support from outside the family (Lanouette et al., 2009)
**EMPLOYMENT STATUS**		
Unemployment (Nosé et al., 2003; Hodari et al., 2006) White-collar employment (Jindal et al., 2003)		Employment status (Karamanidou et al., 2008)

Abbreviations *TB* *tuberculosis;*

P *, determinant of persistence*.

**Table 4:** **Healthcare team and system-related factors affecting adherence**.

**Factors having**		
**Negative effect on adherence**	**Positive effect on adherence**	**Neutral effect on adherence**
**BARRIERS TO HEALTHCARE**		
Barriers to high-quality care (Lanouette et al., 2009) Lack of providers/caregiver availability (Charach and Gajaria, 2008^P^; Vreeman et al., 2008) Rural settings (Vreeman et al., 2008) Poor access to a health care facility (e.g., long waiting times, queues, lack of privacy, inconvenient appointment times, inconvenient opening hours) (Munro et al., 2007) Seeing different language speaking therapist (ie Spanish-speaking therapist in US Latinos) (Lanouette et al., 2009) Difficulty in obtaining sick leave for treatment (Munro et al., 2007) Having no time to refill prescriptions, or other pharmacy-related problems (Mills et al., 2006)	Good access to medication and health service (Fogarty et al., 2002) Good access to a health care facility (Nosé et al., 2003; Munro et al., 2007) Non-emergency referral (Pampallona et al., 2002) Obtaining certification of preventive treatment (for immigrants to US) (Munro et al., 2007)	Access to care (Lacro et al., 2002) Greater distance from the clinic (Jindal et al., 2003; Munro et al., 2007) Current inpatient status (Lacro et al., 2002) Rural settings (vs. urban) (Lacro et al., 2002) Type of transportation used (Lacro et al., 2002)
**DRUG SUPPLY**		
Poor drug supply (e.g., poor TB medication availability at health care facilities) (Mills et al., 2006; Munro et al., 2007) Unavailability of medications (e.g., prescription ran out) (Vik et al., 2004)	Receiving treatment together with methadone from a street nurse (for DOT in TB, in IDU patients) (Munro et al., 2007)
**PRESCRIPTION BY A SPECIALIST**		
	Referral/prescription by a specialist (Pampallona et al., 2002; Van Der Wal et al., 2005)	Prescription by a psychiatrist (in depression) (Lanouette et al., 2009)
**INFORMATION ABOUT DRUG ADMINISTRATION**		
Unclear information about proper drug administration (Vik et al., 2004) Greater number of prescribing physicians (Vik et al., 2004) Conflicting messages between gps and specialists on medication (Hodari et al., 2006) Discrepancies between treatment guidelines and common clinical practice (as patients try to ask several specialists) (Oehl et al., 2000) Use of multiple pharmacies (Vik et al., 2004)	Doctor's ability to provide appropriate information as to the drug administration (Vermeire et al., 2001; Weiner et al., 2008) Being given information about the action of the drugs (Olthoff et al., 2005)	
**HEALTHCARE PROVIDER-PATIENT COMMUNICATION AND RELATIONSHIP**		
Poor healthcare provider-patient relationship (Oehl et al., 2000; Vermeire et al., 2001; Lacro et al., 2002; Nosé et al., 2003; Vik et al., 2004; Olthoff et al., 2005; Hodari et al., 2006; Munro et al., 2007; Charach and Gajaria, 2008^P^; Costello et al., 2008; Broekmans et al., 2009; Julius et al., 2009) Poor patient–physician communication (Vermeire et al., 2001; Gold et al., 2006^P^; Hodari et al., 2006; Munro et al., 2007; Broekmans et al., 2009; Jacobsen et al., 2009; Julius et al., 2009) Lack of trust in doctors and healthcare (Chia et al., 2006; Mills et al., 2006; Broekmans et al., 2009) Lack of patient satisfaction with their healthcare, (Hodari et al., 2006; Mills et al., 2006) Limited caregiver adherence strategies (Vreeman et al., 2008)	Quality, duration and frequency of interaction between the patient and doctor (Vermeire et al., 2001) Offering enough time to the patient, leaving space to talk about problems concerning medication or side effects (Oehl et al., 2000) Patient involvement in decision making (Gold et al., 2006^P^; Mills et al., 2006; Ruddy et al., 2009) Encouraging self-management (Weiner et al., 2008) Doctor responsiveness (Vermeire et al., 2001) Doctor's ability to demonstrate empathy (Vermeire et al., 2001) Doctor's ability to elicit and respect the patient's concerns (Vermeire et al., 2001) Perceived healthcare provider support (Fogarty et al., 2002; Costello et al., 2008)	
**FOLLOW-UP**		
Inadequate discharge planning (Julius et al., 2009; Lacro et al., 2002) Fewer outpatient visits (Vik et al., 2004; Olthoff et al., 2005; Van Der Wal et al., 2005; Broekmans et al., 2009; Julius et al., 2009) Poor follow-up by providers (Lacro et al., 2002; Gold et al., 2006^P^; Munro et al., 2007; Reisner et al., 2009)	More visits to a nonmedical therapist (Lanouette et al., 2009) Seeing a greater number of physicians (Ruddy et al., 2009)	Clinic attendance (Jindal et al., 2003)

Abbreviations *GP* *general practitioner;* *TB* *tuberculosis;*

* *, determinant of persistence*.

**Table 5:** **Condition-related factors affecting adherence**.

**Factors having**		
**Negative effect on adherence**	**Positive effect on adherence**	**Neutral effect on adherence**
**PRESENCE OF SYMPTOMS**		
Asymptomatic nature of the disease or absence of symptoms (Vermeire et al., 2001; Olthoff et al., 2005; Gold et al., 2006^P^; Costello et al., 2008)	Increased severity and number of symptom (Nosé et al., 2003; Munro et al., 2007; Charach and Gajaria, 2008^P^; Brandes et al., 2009; Lanouette et al., 2009) Disability (Vermeire et al., 2001; Costello et al., 2008)	Pain duration (Broekmans et al., 2009) Pain intensity (Broekmans et al., 2009) Presence of tremor (Jindal et al., 2003)
**DISEASE SEVERITY**		
Lower affective pain ratings (Broekmans et al., 2009) Detectable viral load (in HIV-infected youth) (Reisner et al., 2009)	Disease severity (Van Der Wal et al., 2005; DiMatteo et al., 2007; Reisner et al., 2009; Ruddy et al., 2009) Perceptions of disease severity (DiMatteo et al., 2007) More hospitalization (before starting ART in children) (Vreeman et al., 2008)	Disease severity (Cramer, 2004; DiMatteo, 2004b; Chia et al., 2006; DiMatteo et al., 2007; Weiner et al., 2008; Julius et al., 2009; Lanouette et al., 2009) Worse clinical status (Fogarty et al., 2002) Possible consequences of missed doses (Cramer, 2004)
**CLINICAL IMPROVEMENT**		
Clinical improvement, disappearance of symptoms, feeling better/cured (Oehl et al., 2000^P^; Mills et al., 2006; Munro et al., 2007^P^; Ruddy et al., 2009) Onset of clinical symptoms (in latent TB infection) (Hirsch-Moverman et al., 2008)	Perception of a clinical improvement (Oehl et al., 2000) Reduced viral load (in HIV-infected youth) (Reisner et al., 2009)	
**PSYCHIATRIC CONDITION**		
Psychiatric disorders (Vermeire et al., 2001; Nosé et al., 2003) Negative symptoms/motivational deficits (Oehl et al., 2000)	Lower rates of narcissistic-histrionic personality disorder (in depression) (Pampallona et al., 2002)	Severity of psychotic symptoms (Lacro et al., 2002)
**CERTAIN DIAGNOSES/INDICATIONS**		
Certain diagnoses (pulmonary conditions, DM, and sleep disorders vs. other) (DiMatteo, 2004b) Indication (pain medication vs. other medications) (Broekmans et al., 2009)	Certain diagnoses: rheumatoid arthritis vs. other types of arthritis (Broekmans et al., 2009), combined subtype in ADHD, vs. inattentive or hyperactive subtype (Charach and Gajaria, 2008^P^), disease group (HIV, arthritis, GI diseases, and cancer vs. other) (DiMatteo, 2004b), disease group (diagnosis other than personality disorder and substance abuse, in depression) (Pampallona et al., 2002) Estrogen receptor positive (in breast cancer) (Ruddy et al., 2009)	Cause of ESRD (Karamanidou et al., 2008) Latent or active TB (Munro et al., 2007) Disease factors (Vermeire et al., 2001)
**DURATION OF THE DISEASE**		
Chronic nature of the disease (Hodari et al., 2006) Longer time since clinic visit (Olthoff et al., 2005) Longer time since transplant (Jacobsen et al., 2009) Later disease stage (in HIV-infected youth) (Reisner et al., 2009) Shorter duration of illness (in schizophrenia) (Lacro et al., 2002)	Longer duration of pain (Chia et al., 2006)	Duration of the disease (Lanouette et al., 2009) Length of time of hemodialysis (Karamanidou et al., 2008; Schmid et al., 2009)

Abbreviations *ART* *antiretroviral therapy;* *ESRD* *end stage renal disease;* *TB* *tuberculosis;*

P *, determinant of persistence*.

**Table 6:** **Therapy-related factors affecting adherence**.

**Factors having**		
**Negative effect on adherence**	**Positive effect on adherence**	**Neutral effect on adherence**
**ADVERSE EFFECTS**		
Adverse effects (Oehl et al., 2000; Vermeire et al., 2001; Fogarty et al., 2002; Pampallona et al., 2002; Vik et al., 2004; Chia et al., 2006; Gold et al., 2006^P^; Hodari et al., 2006; Mills et al., 2006; Lewiecki, 2007; Munro et al., 2007; Charach and Gajaria, 2008^P^; Costello et al., 2008^P^; Karamanidou et al., 2008; Vreeman et al., 2008; Weiner et al., 2008; Brandes et al., 2009^P^; Julius et al., 2009; Reisner et al., 2009; Schmid et al., 2009) Decreased quality of life while taking medications (Hodari et al., 2006; Mills et al., 2006)		Adverse effects (Lacro et al., 2002; Olthoff et al., 2005)
**PATIENT FRIENDLINESS OF THE REGIMEN**		
Complexity of the regimen (e.g., complex/frequent dosing schedule/number of tablets) (Oehl et al., 2000; Vermeire et al., 2001; Fogarty et al., 2002; Van Der Wal et al., 2005; Gold et al., 2006^P^; Mills et al., 2006; Munro et al., 2007^P^; Vreeman et al., 2008; Weiner et al., 2008; Brandes et al., 2009; Julius et al., 2009; Schmid et al., 2009) Dosing frequency (Claxton et al., 2001; Vermeire et al., 2001; Olthoff et al., 2005; Van Der Wal et al., 2005; Hodari et al., 2006; Mills et al., 2006; Charach and Gajaria, 2008^P^; Vreeman et al., 2008) Number of prescribed medications (polymedication) (Vermeire et al., 2001; Broekmans et al., 2009) Less medication prescribed (in patients with chronic non-malignant pain) (Broekmans et al., 2009) Doses during day (particularly the middle-of-day or early-morning doses) (Mills et al., 2006; Charach and Gajaria, 2008^P^) Instability of the regimen (Van Der Wal et al., 2005) Inconvenience associated with administration of some medication (e.g., oral biphosphonates) (Olthoff et al., 2005; Gold et al., 2006^P^; Hodari et al., 2006; Brandes et al., 2009) Injection formulation (e.g., insulin) (Cramer, 2004; Munro et al., 2007^P^; Costello et al., 2008; Brandes et al., 2009) Need to adjust dietary habits for taking medication (Fogarty et al., 2002; Hodari et al., 2006; Mills et al., 2006; Munro et al., 2007; Vreeman et al., 2008) Problems with opening containers (Vik et al., 2004) Disliking aspects of the medication (Ruddy et al., 2009) Poor taste of medication (Mills et al., 2006; Weiner et al., 2008; Schmid et al., 2009) Big tablet size, problems with swallowing tablets (Vik et al., 2004; Mills et al., 2006; Weiner et al., 2008; Schmid et al., 2009)	Once-daily dosing (vs. more frequent one) (Iskedjian et al., 2002; Cramer, 2004; Wetzels et al., 2004; Lee et al., 2006; Parienti et al., 2009) Once-weekly dosing (vs. once-daily) (Kruk and Schwalbe, 2006) Simple regimen (Mills et al., 2006) Fewer drugs prescribed (Cramer, 2004; Reisner et al., 2009) Fixed-dose combination pills (Connor et al., 2004; Yeung and White, 2005) Long acting formulation (Charach and Gajaria, 2008^P^) Unit-of-use packaging (Connor et al., 2004) Flexibility/patient choice in treatment (Munro et al., 2007; Bao et al., 2009^P^) Dosing through injections (Oehl et al., 2000; Vermeire et al., 2001; Lewiecki, 2007; Schmid et al., 2009) Regular medication schedule (vs. irregular dose interval) (Van Der Wal et al., 2005)	Simplicity of regimen (Cramer, 2004) Regimen complexity (Lacro et al., 2002; Olthoff et al., 2005; Karamanidou et al., 2008) Number of prescribed medications (Chia et al., 2006) Once-monthly dosing (vs. once-daily) (Kruk and Schwalbe, 2006) Route of medication administration (Lacro et al., 2002) Use of oral medication (vs. depot ones) (Lacro et al., 2002)
**DRUG EFFECTIVENESS**		
Drug ineffectiveness, objective, or perceived (Oehl et al., 2000; Vik et al., 2004; Munro et al., 2007^P^; Charach and Gajaria, 2008^P^; Costello et al., 2008^P^; Brandes et al., 2009)	Relief of symptoms (Munro et al., 2007^P^; Weiner et al., 2008) Objective drug effectiveness (Yeung and White, 2005; Mills et al., 2006; Costello et al., 2008)	
**DURATION OF THE TREATMENT**		
Longer duration of treatment (Vermeire et al., 2001; Wetzels et al., 2004; Munro et al., 2007^P^; Vreeman et al., 2008; Reisner et al., 2009)	Shorter duration of treatment (Hirsch-Moverman et al., 2008)	Duration of treatment (Ruddy et al., 2009)
**DRUG TYPE**		
Drug type (olanzapine vs. risperidon) (Santarlasci and Messori, 2003^P^) Higher antipsychotic dose (Lacro et al., 2002)	Drug class (aRB vs. ACEi, BBs, CCBs, diuretics) (Bramlage and Hasford, 2009^P^) Drug type (fluoxetine, nortriptiline, or imipramine, vs. other antidepressants) (Pampallona et al., 2002), (fluoxetine vs. others) (Pampallona et al., 2002; Lanouette et al., 2009^P^) Boosted protease inhibitors (vs. standard therapy) (Ramos, 2009) Greater methadone doses (Bao et al., 2009^P^)	Class of medication (Lacro et al., 2002; Julius et al., 2009) Dose of prednisone (Jindal et al., 2003) Type of treatment program (in TB) (Munro et al., 2007)
**WELL ORGANISED TREATMENT**		
	Receiving care in structured settings (e.g., DOT) (Malta et al., 2008) Treatment at medical center (Charach and Gajaria, 2008^P^) Well-structured treatment plan (Oehl et al., 2000) Psychotherapy (along with psychotropic medication) (Lanouette et al., 2009)	Medication supervision status (Lacro et al., 2002) Having a case manager (Lacro et al., 2002) Being aware of monitoring (Wetzels et al., 2004)

Abbreviations *ACEi* *angiotensin-converting-enzyme inhibitors;* *aRB* *angiotensin II receptor antagonists;* *BBs* *beta-blockers;* *CCBs* *calcium channel blockers;* *DOT* *directly observed therapy;*

P *, determinant of persistence*.

**Table 7:** **Patient-related factors affecting adherence**.

**Factors having**		
**Negative effect on adherence**	**Positive effect on adherence**	**Neutral effect on adherence**
**AGE**		
Younger age (Fogarty et al., 2002; Jindal et al., 2003; Nosé et al., 2003; Van Der Wal et al., 2005; Chia et al., 2006; Karamanidou et al., 2008; Julius et al., 2009; Lanouette et al., 2009; Ruddy et al., 2009; Schmid et al., 2009) Older children (vs. younger ones) (Weiner et al., 2008) Age - older and younger age groups (vs. adults) (Munro et al., 2007) Very old age (older than 85 years) (Ruddy et al., 2009)	Younger females (vs. older ones) (Oehl et al., 2000)	Age (Oehl et al., 2000; Vermeire et al., 2001; Lacro et al., 2002; DiMatteo, 2004b; Vik et al., 2004; Olthoff et al., 2005; Hodari et al., 2006; Hirsch-Moverman et al., 2008; Reisner et al., 2009; Ruddy et al., 2009)
**GENDER**		
Male gender (Oehl et al., 2000; Pampallona et al., 2002; Nosé et al., 2003; Olthoff et al., 2005; Chia et al., 2006; Munro et al., 2007; Julius et al., 2009; Schmid et al., 2009)	Male gender (Jindal et al., 2003; Charach and Gajaria, 2008^P^)	Gender (Vermeire et al., 2001; Fogarty et al., 2002; Lacro et al., 2002; DiMatteo, 2004b; Vik et al., 2004; Van Der Wal et al., 2005; Charach and Gajaria, 2008^P^; Hirsch-Moverman et al., 2008; Karamanidou et al., 2008; Broekmans et al., 2009; Lanouette et al., 2009; Reisner et al., 2009)
**MARITAL STATUS**		
Single or divorced (vs. married) (Jindal et al., 2003; Julius et al., 2009) Being married (in psychosis) (Nosé et al., 2003)	Being married (Pampallona et al., 2002; DiMatteo, 2004a; Hodari et al., 2006; Lanouette et al., 2009) Living with someone (vs. living alone) (DiMatteo, 2004a) Living alone/being single (in psychosis) (Nosé et al., 2003)	Marital status (Vermeire et al., 2001; Lacro et al., 2002; Vik et al., 2004; Karamanidou et al., 2008) Orphan status (Vreeman et al., 2008)
**EDUCATION**		
Illiteracy (Munro et al., 2007) Having repeated a grade in school (in HIV-infected youth) (Reisner et al., 2009)	Education (Pampallona et al., 2002; Nosé et al., 2003; DiMatteo, 2004b; Munro et al., 2007; Hirsch-Moverman et al., 2008; Julius et al., 2009; Schmid et al., 2009) Being in school (vs. not being, in HIV-infected youth) (Reisner et al., 2009) High IQ (Pampallona et al., 2002)	Education (Lacro et al., 2002; Vik et al., 2004; Olthoff et al., 2005; Van Der Wal et al., 2005; Karamanidou et al., 2008; Broekmans et al., 2009; Lanouette et al., 2009)
**ETHNICITY**		
Latinos (vs. Euro-Americans) (Lanouette et al., 2009) Hispanic patients (in the US, in TB) (Munro et al., 2007) Monolingual Spanish speakers (Lanouette et al., 2009) Non-white women (Ruddy et al., 2009)	Caucasian race (Jindal et al., 2003; Charach and Gajaria, 2008^P^) U.S. born (Jindal et al., 2003)	Ethnicity (Lacro et al., 2002; Vik et al., 2004; Van Der Wal et al., 2005; Hirsch-Moverman et al., 2008; Karamanidou et al., 2008; Reisner et al., 2009; Schmid et al., 2009) Place of birth (Hirsch-Moverman et al., 2008)
**HOUSING**		
Unstable housing (Hirsch-Moverman et al., 2008; Julius et al., 2009; Reisner et al., 2009) Homelessness (Mills et al., 2006) Residentially mobile (Munro et al., 2007) Being away from home (Mills et al., 2006; Karamanidou et al., 2008; Vreeman et al., 2008; Schmid et al., 2009)	Structured environment away from home (Munro et al., 2007)	Homelessness (Munro et al., 2007; Hirsch-Moverman et al., 2008) Living arrangements (Lacro et al., 2002; Vik et al., 2004; Lanouette et al., 2009)
**COGNITIVE FUNCTION**		
Cognitive impairment, low attention and working memory (Fogarty et al., 2002; Nosé et al., 2003; Lovejoy and Suhr, 2009; Schmid et al., 2009)		Neurocognitive impairment (Lacro et al., 2002; Lovejoy and Suhr, 2009) Verbal fluency (Lovejoy and Suhr, 2009)
**FORGETFULNESS AND REMINDERS**		
Forgetting (Fogarty et al., 2002; Vik et al., 2004; Mills et al., 2006; Schmid et al., 2009; Weiner et al., 2008) Sleeping through a dose (Mills et al., 2006)	Making use of reminders (Mills et al., 2006; Munro et al., 2007) Using friends and family as reminders (Mills et al., 2006) Having a routine in which taking drugs could be easily incorporated (Mills et al., 2006)	
**KNOWLEDGE**		
Lack of comprehension of disease and treatment (Vermeire et al., 2001; Olthoff et al., 2005; Gold et al., 2006^P^; Lewiecki, 2007; Charach and Gajaria, 2008^P^; Karamanidou et al., 2008; Vreeman et al., 2008) Misunderstanding of the prescription and treatment instructions, and the consequences of non-adherence (Vik et al., 2004; Mills et al., 2006; Munro et al., 2007; Vreeman et al., 2008) Misconceptions reported from the media, lay press, family or friends, about a medication (Hodari et al., 2006) Obtaining helpful breast cancer information from books or magazines (in breast cancer) (Ruddy et al., 2009)	Situational operational knowledge (Jindal et al., 2003; Mills et al., 2006) Understanding the need for strict adherence (Mills et al., 2006)	
**HEALTH BELIEFS**		
Denial of diagnosis (Vermeire et al., 2001; Munro et al., 2007) Unrealistic expectations concerning the medication's benefit/risk ratio (Oehl et al., 2000) Negative patients' beliefs about the efficacy of treatment (Mills et al., 2006; Munro et al., 2007; Malta et al., 2008; Weiner et al., 2008; Reisner et al., 2009) Negative attitude toward or subjective response to medication (Lacro et al., 2002) Thinking that the treatment could make the patients ill (Munro et al., 2007) Belief that taking medication together with concurrent western or traditional medicines may have negative consequences (in TB) (Munro et al., 2007) Belief that pregnancy would increase intolerance to drugs and make TB drugs ineffective (Munro et al., 2007) Concerns that the treatment would affect immigration status, and lead to disclosure of illegal immigrant status/incarceration (in TB) (Munro et al., 2007) Having doubts, or not being able to accept HIV status (Mills et al., 2006) Unresolved concerns about time between taking the drug and its effect (Vermeire et al., 2001) Being suspicious of treatment/medical establishment (Mills et al., 2006) Interpreting DOT as distrust (Munro et al., 2007) “Being tired” of taking medications (Munro et al., 2007^P^) Feeling that treatment is a reminder of HIV status (Mills et al., 2006) Perceived excessive medication use (Vik et al., 2004) Feeling persecuted or poisoned (Oehl et al., 2000) Lack of interest in treatment (Munro et al., 2007) Wanting to be free of medications or preferring a natural approach (Mills et al., 2006) Wanting to be in control (Mills et al., 2006) Prioritizing work over taking treatment (Munro et al., 2007)	Belief in the diagnosis (Vermeire et al., 2001) Belief in a particular set of health recommendations (Vermeire et al., 2001) Belief in self-efficacy for taking medication (Chia et al., 2006) Self-confidence to maintain health status (Van Der Wal et al., 2005) Fewer concerns about drugs, belief that medication is safe (Chia et al., 2006; Charach and Gajaria, 2008^P^) Belief that asthma is not caused by the external factors (Chia et al., 2006) Lower belief in natural products and home remedies (Chia et al., 2006) Beliefs of control over one's health (Chia et al., 2006) Feeling of empowerment (Brandes et al., 2009) Lower control beliefs about cancer-related pain (Chia et al., 2006) Perceived benefits of adherence (Chia et al., 2006; Munro et al., 2007; Costello et al., 2008; Hirsch-Moverman et al., 2008; Karamanidou et al., 2008) Desire to avoid burdening family members (Costello et al., 2008) More motivation (Lanouette et al., 2009) Belief that they are vulnerable or susceptible to the disease or its consequences (Vermeire et al., 2001) Worrying about the disease (Weiner et al., 2008) Perceived the necessity of treatment (Chia et al., 2006; Hirsch-Moverman et al., 2008) Regarding drugs as vital (as opposed to important) (Olthoff et al., 2005) Felt less burdened by taking the medication (Chia et al., 2006) Fear of experiencing relapses and future disability (Costello et al., 2008)	HIV disease attitudes (Fogarty et al., 2002) Feeling invulnerable to the consequences of HIV (Reisner et al., 2009)
**PSYCHOLOGICAL PROFILE**		
Personality: low conscientiousness, high cynical hostility (Karamanidou et al., 2008) Pessimistic ways of coping (Weiner et al., 2008) Withdrawal coping style, or self-destructive escape coping style (Reisner et al., 2009) Poor insight (Lacro et al., 2002) Lack of self-worth (Mills et al., 2006) Oppositional behaviours (Weiner et al., 2008) Laziness/lack of care (Munro et al., 2007) Being too distracted or busy (Mills et al., 2006)	Accepting the HIV-seropositivity (Mills et al., 2006) Coping psychologically with HIV diagnosis (Munro et al., 2007) Optimistic ways of coping (Weiner et al., 2008) Hope (Costello et al., 2008) Insight (Nosé et al., 2003) Higher self-efficacy (Jindal et al., 2003; Costello et al., 2008; Reisner et al., 2009) Higher levels of life satisfaction (Reisner et al., 2009) Internal locus of control (Schmid et al., 2009) Self-esteem (Mills et al., 2006; Costello et al., 2008) Lower levels of psychologic distress (Reisner et al., 2009) Personal control of the disease and therapy (Costello et al., 2008; Weiner et al., 2008) Higher level of self-care agency score (Jindal et al., 2003) Living for someone, especially, children (Mills et al., 2006) Rewarding oneself after injections (Costello et al., 2008)	Coping style (Karamanidou et al., 2008) Emotional overinvolvement (Lanouette et al., 2009) Warmth (Lanouette et al., 2009) More insight (Lanouette et al., 2009) Criticism (Lanouette et al., 2009) Less busy lifestyle (Chia et al., 2006) Problems with role functioning (Lanouette et al., 2009)
**COMORBIDITIES AND PATIENT HISTORY**		
Having other concurrent illnesses affecting adherence (Mills et al., 2006) Non-adherence in the past (Lacro et al., 2002; Nosé et al., 2003) Previous treatment failure (Hodari et al., 2006) Concurrent diseases or illnesses, including malnutrition (Mills et al., 2006) Psychiatric illness, e.g., anxiety/depression (Jindal et al., 2003; Nosé et al., 2003; Hodari et al., 2006; Mills et al., 2006; Munro et al., 2007; Karamanidou et al., 2008; Malta et al., 2008; Reisner et al., 2009; Schmid et al., 2009) Prior suicide attempt (Reisner et al., 2009) Concomitant medication use (in latent TB) (Hirsch-Moverman et al., 2008) Recent hospitalization (Hirsch-Moverman et al., 2008) Long hospital stay (Nosé et al., 2003) Higher number of transplants and rejection episodes (Jindal et al., 2003) Both eye blindness (Olthoff et al., 2005) Impaired motor functioning (Lovejoy and Suhr, 2009) History of infection (in patients after kidney transplantation) (Jindal et al., 2003) No history of diabetes (Jindal et al., 2003) Sexual abuse under age of 12 years (Reisner et al., 2009) Recent incarceration (Malta et al., 2008) Receiving standard primary tumour therapy (in tamoxifen use in breast cancer) (Ruddy et al., 2009^P^)	Less chronic co-morbidities (Van Der Wal et al., 2005) More severe comorbid conditions (Charach and Gajaria, 2008)^P^ No previous use of disease modifying therapies (in MS) (Costello et al., 2008) Previous psychiatric contacts (in patients with psychosis (Nosé et al., 2003) Previous use of antidepressants (in depression) (Pampallona et al., 2002) Witnessing the consequences of not following medical advice in relatives with other diseases (Costello et al., 2008) Prior history of treatment with stimulants (in ADHD) (Charach and Gajaria, 2008)^P^ Current psychiatric treatment (in depression) (Pampallona et al., 2002) Being less likely to have bartered sex during the lifetime (in HIV-infected youth) (Reisner et al., 2009) Being less likely to have had a sexually transmitted disease since learning their serostatus (in HIV-infected youth) (Reisner et al., 2009) Using condoms with recent sex partners (in HIV-infected youth) (Reisner et al., 2009) Diagnosis of asthma or COPD (in HF patients) (Van Der Wal et al., 2005) Lack of relapse (in depression) (Pampallona et al., 2002) Recent exposure to TB (Hirsch-Moverman et al., 2008) Previous readmission for all causes (in HF) (Van Der Wal et al., 2005) Previous readmission for HF (in HF) (Van Der Wal et al., 2005)	Number of medical conditions (Chia et al., 2006) Adherence to other parts of an inpatient treatment program (Lacro et al., 2002) Presence of mood symptoms (or diagnosis of schizoaffective or bipolar disorder) (Lacro et al., 2002) Anxiety (DiMatteo et al., 2007) Concurrent methadone treatment (in latent TB infection) (Hirsch-Moverman et al., 2008) Total number of therapists in lifetime (Lanouette et al., 2009) Number of medications prescribed for another condition (Olthoff et al., 2005) Diabetes, as a comorbidity (Karamanidou et al., 2008) Dialysis compliance (Jindal et al., 2003) Type of the dialysis (Karamanidou et al., 2008) Patient's transplant history (Kahana et al., 2008; Karamanidou et al., 2008) Donor/graft source (Jindal et al., 2003; Kahana et al., 2008) Treated rejection episodes (Jindal et al., 2003)
**ALCOHOL OR SUBSTANCE ABUSE**		
Substance abuse (Oehl et al., 2000; Lacro et al., 2002; Nosé et al., 2003; Mills et al., 2006; Munro et al., 2007; Malta et al., 2008; Lanouette et al., 2009) Injection drugs use (vs. non-injection ones) (Malta et al., 2008) Younger age of first marijuana use (Reisner et al., 2009) Alcohol abuse (Oehl et al., 2000) Smoking (Hodari et al., 2006; Schmid et al., 2009)	Less recent drug use in the previous 3 months (in HIV-infected youth) (Reisner et al., 2009) Medication taking priority over substance use (Mills et al., 2006) Drug addiction treatment, especially substitution therapy (for HIV treatment in drug users) (Malta et al., 2008) Drinking less, or non-drinking (Hodari et al., 2006; Reisner et al., 2009)	Injective drug using (Munro et al., 2007)
**PATIENT-RELATED BARRIERS TO COMPLIANCE**		
Transportation difficulties (Mills et al., 2006; Schmid et al., 2009)		

Abbreviations *HF* *heart failure;* *MS* *multiple sclerosis;* *TB* *tuberculosis;*

P *, determinant of persistence*.

## Discussion

In this systematic literature review, 51 systematic reviews concerning the determinants of adherence of medication were identified. Remarkably, a vast majority of the reviewed literature provided only determinants of implementation. In fact, many reviews lacked a clear definition of adherence, thus leaving the distinction between implementation and persistence open to interpretation. In the present study, these cases were arbitrarily reclassified under determinants of implementation, assuming that in most cases, authors were interested in the day-to-day execution of drug taking. The recently-agreed European consensus on taxonomy and terminology of adherence has made more precise reporting of research findings in the field of adherence to medication possible (Vrijens et al., 2012). However, in interpreting results of this study, one has to have in mind this limitation.

Many reviews reported a positive effect of family and social support on implementation, and a negative effect of the lack of such support (Table 3). The social stigma of a disease may also be responsible for non-adherence in a number of cases. Finally, economic factors such as unemployment, poverty, lack of, or inadequate medical/prescription coverage, as well as a high out-of-pocket cost of drugs may seriously contribute to non-adherence.

Although non-adherence has often been perceived as the fault of patients, and not of healthcare providers, there is evidence that healthcare system factors have an important impact on adherence (Table 4). Poor access to healthcare, poor drug supply, unclear information about drug administration, as well as poor follow-up and poor provider-patient communication and relationship may reduce the extent to which patients follow the treatment plan.

Adherence is also related to condition. Asymptomatic nature of the disease, as well as clinical improvement reduce patient motivation to take the drugs as prescribed, whereas disease severity has a positive effect on adherence (Table 5). Patients are also less happy to take the prescribed medication properly in both chronic and psychiatric conditions.

If treatment is patient unfriendly – e.g., due to frequent dosing, high number of prescribed medications, longer duration of treatment, drug formulation or taste of low acceptance, or the presence of adverse effects, the likelihood of patient adherence drops (Table 6). Certain drug classes are better adhered to compared with others.

Not surprisingly, many patient-related factors were found to be reported as having an inconsistent impact on adherence in terms of implementation (Table 7). This was particularly true for demographic factors: whereas younger age was reported to have a negative impact on adherence, and older age a positive one, many reviews found no relationship between age and implementation of treatment regimen (Oehl et al., 2000; Vermeire et al., 2001; Lacro et al., 2002; DiMatteo, 2004b; Vik et al., 2004; Olthoff et al., 2005; Hodari et al., 2006; Hirsch-Moverman et al., 2008; Reisner et al., 2009; Ruddy et al., 2009). The male gender was reported to have a negative impact in some reviews (Nosé et al., 2003; Olthoff et al., 2005; Schmid et al., 2009), and the female gender a positive one (Oehl et al., 2000; Pampallona et al., 2002; Chia et al., 2006; Munro et al., 2007; Julius et al., 2009). However, gender was found irrelevant for adherence in many cases (Vermeire et al., 2001; Fogarty et al., 2002; Lacro et al., 2002; DiMatteo, 2004b; Vik et al., 2004; Van Der Wal et al., 2005; Charach and Gajaria, 2008; Hirsch-Moverman et al., 2008; Karamanidou et al., 2008; Broekmans et al., 2009; Lanouette et al., 2009; Reisner et al., 2009), and male gender was found to have a contrary effect with posttransplant medications (Charach and Gajaria, 2008) and with psychostymulants in children with ADHD (Jindal et al., 2003). The same was true for marital status, with some reviews indicating that those married tended to have better adherence than those being single or divorced, education level, with better adherence demonstrated by patients with higher levels of education, and ethnicity, with higher adherence in Caucasians. Patient attitudes and believes in favor of diagnosis, health recommendations and self-efficacy were closely related to adherence, as was knowledge of the disease and consequences of poor adherence. On the other hand, many beliefs were found to be possible barriers for strict adherence. Poorer adherence can be expected with either drug or alcohol dependence. Finally, comorbidities and patient history had an inconsistent effect on adherence, with the exception of psychiatric conditions, which was frequently reported to be connected with the lower rates of adherence (Claxton et al., 2001; Jindal et al., 2003; Nosé et al., 2003; Hodari et al., 2006; Mills et al., 2006; Munro et al., 2007; Charach and Gajaria, 2008; Karamanidou et al., 2008; Malta et al., 2008; Reisner et al., 2009; Schmid et al., 2009).

Only few determinants of persistence were identified. Socio-economic factors with a negative impact on persistence included high costs of drugs and treatment (Gold et al., 2006; Munro et al., 2007; Costello et al., 2008), poverty (Costello et al., 2008), lower socioeconomic status (Charach and Gajaria, 2008), or inadequate medical/prescription coverage (Charach and Gajaria, 2008; Costello et al., 2008). Several healthcare system-related factors also had a negative effect on persistence, such as lack of providers/caregiver availability (Charach and Gajaria, 2008), poor healthcare provider-patient relationship (Charach and Gajaria, 2008), or poor follow-up by providers (Gold et al., 2006). Asymptomatic nature of disease (Gold et al., 2006), as well as clinical improvement, disappearance of symptoms, feeling better/cured (Oehl et al., 2000; Munro et al., 2007), the presence of adverse effects (Gold et al., 2006; Charach and Gajaria, 2008; Costello et al., 2008; Brandes et al., 2009) and complexity of the regimen (Gold et al., 2006; Munro et al., 2007) all decreased patient motivation to persist with treatment, as did high dosing frequency (Charach and Gajaria, 2008), doses during the day (Charach and Gajaria, 2008), and finally, drug ineffectiveness, objective or perceived (Munro et al., 2007; Charach and Gajaria, 2008; Costello et al., 2008). This findings are of special interest, as longer persistence is a primary goal for adherence-enhancing interventions. On the other hand, it is noteworthy that the vast majority of persistence determinants were also implementation determinants (see Tables 3–7).

Our findings are consistent with those of the other authors (Vermeire et al., 2001; DiMatteo, 2004b). However, the strength of this study is the rigorous methodology that we employed to classify literature search findings. A predefined set of criteria, and the use of well-defined terminology to describe the deviation of patients from prescribed treatment allowed a cohesive matrix of factors to be built that were determinants of either adherence or non-adherence. Bearing in mind that at least 200 factors have so far been suggested to play some role in determining adherence (Vermeire et al., 2001), the approach adopted in our study seems to move our understanding of adherence to medication forward. The clear distinction between *implementation* of the regimen (daily drug-taking) and *persistence* (continuity of treatment) allows the first, to the best of our knowledge, clear distinction of the determinants of these two components of adherence to medication to be made, thus providing a more detailed insight into the role of some determinants of the adherence process, compared with previous approaches (e.g., the WHO 5 dimensions).

Our analysis provides clear evidence that medication non-adherence is affected by multiple determinants, belonging to several different fields. Many of these factors are not modifiable, and none of them is a sole predictor of adherence. Moreover, some of these factors change with time and can appear at times either to be a cause, or a consequence, of patient non-adherence. Nevertheless, non-adherence should not be perceived as patients' fault only. To the contrary, social factors (such as social support, economic factors, etc.), healthcare-related factors (e.g., barriers to healthcare, and quality of provider-patient communication), condition characteristics, as well as therapy-related factors (such as patient friendliness of the therapy) play an important role in defining adherence.

Consequently, multifaceted interventions may be the most effective answer toward unsatisfactory adherence, and its consequences. In their review of reviews of effectiveness of adherence-enhancing interventions, van Dulmen et al. found effective interventions in each of four groups: technical, behavioral, educational and multi-faceted or complex interventions (van Dulmen et al., 2007). In their Cochrane review, Haynes et al. (Haynes et al., 2008) observed that most of the interventions that were effective for long-term care were complex, targeting multiple adherence determinants. We believe that evidence accumulated in this study may help in designing such effective interventions, and thus, be applied in both clinical practice and public health.

Bearing in mind the number of identified determinants and their inconsistent effect on adherence, prediction of non-adherence of individual patients is difficult if not impossible. In particular, the inconsistent effect of demographic variables on patient adherence explains partly why healthcare providers are ineffective in predicting adherence in their patients (Okeke et al., 2008). In fact, their prediction rate is no better than a coin toss (Mushlin and Appel, 1977). Neither age, gender, marital status, nor education proved to fully explain the variance in patient adherence across conditions and settings. Therefore, in order to reveal cases of non-adherence, validated tools (e.g., Morisky, or MARS questionnaires), and objective assessment methods (electronic monitoring widely accepted as a gold standard) are strongly advisable (Osterberg and Blaschke, 2005). In daily practice, relevant databases, such as electronic health records, and pharmacy fill records, may be effectively used for screening for non-adherence (Carroll et al., 2011; Grimes et al., 2013). On the other hand, adherence-enhancing interventions are worth considering to implement in daily clinical practice, to be used on a regular basis for every individual patients.

Finally, another strength of this systematic literature review is the identification of existing gaps in our understanding of adherence. Of note is that despite the broad inclusion criteria adopted for this search, no systematic review was identified which provides determinants of adherence with short-term treatments. Considering the high prevalence of non-adherence to short-term therapies, and especially, to antibiotics (Kardas et al., 2005; Vrijens et al., 2005), our findings identify this field as an important area for future research.

The major limitation of this study was connected with the data available within the source publications that were used for this review. Most did not provide any precise definition of adherence, nor any numeric values to describe the effect of the particular determinants on adherence (e.g., the effect size), thus making secondary analysis not manageable. The poor designs of many original studies on determinants of non-adherence could affect the conclusions of identified reviews, and indirectly, the results of this review.

The “review of reviews” methodology we employed in the present study proved to be a valuable tool for gathering relevant studies. However, despite the fact that the source reviews adopted different focuses, the certain level of overlap in primary studies they reviewed cannot be ruled out. Nevertheless, as the aim of the study was to build a comprehensive list of determinants, and not to perform a meta-analysis, this possible overlap was not a source of additional bias.

Finally, although our selection of the databases searched was only arbitral, it did correspond with the major goal, i.e., identification of publications describing determinants of adherence to medication. According to our experience, and knowledge of similar publications, broadening the scope of the databases included would not add much to the findings.

### Conflict of interest statement

The authors declare that the research was conducted in the absence of any commercial or financial relationships that could be construed as a potential conflict of interest.
