# Supplementary material for: Determinants of patient adherence: a review of systematic reviews
Source: Front Pharmacol. 2013 Jul 25;4:91. doi: 10.3389/fphar.2013.00091 (PMC3722478; doi:10.3389/fphar.2013.00091)
Supplement: Supplementary file 1 [file DataSheet1.PDF]

Appendix 1:

Search strategies

A. Search strategy used in MEDLINE (via Pubmed) database

1. patient compliance [majr]
2. patient dropouts [majr]
3. treatment refusal [majr]
4. directly observed therapy [majr]
5. medication adherence [majr]
6. concordance [tiab] AND patient compliance [mh]
7. 1 OR 2 OR 3 OR 4 OR 5 OR 6
8. factor [tw] OR factors [tw]
9. variable [tw] OR variables [tw] OR variable* [tw]
10. predictor [tw] OR predictors [tw] OR predict* [tw]
11. determinant [tw] OR determinants [tw] OR determin* [tw]
12. association [tw] OR associations [tw] OR associat* [tw]
13. 8 OR 9 OR 10 OR 11 OR 12
14. 7 AND 13
15. systematic [tw]
16. 14 AND 15
17. 14 Limits: Systematic Reviews
18. 16 OR 17
19. Limits: Publication date from 2000/01/01 to 2009/12/31
20. Limits: Humans, English

B. Search strategy used in EMBASE

1 exp patient compliance/

2 patients dropouts.mp. [mp=title, abstract, subject headings, heading word, drug trade name, original title, device manufacturer, drug manufacturer name]

3 exp treatment refusal/

4 exp directly observed therapy/

5 medication adherence.mp. [mp=title, abstract, subject headings, heading word, drug trade name, original title, device manufacturer, drug manufacturer name]

6 concordance.mp. [mp=title, abstract, subject headings, heading word, drug trade name, original title, device manufacturer, drug manufacturer name]

7 1 or 2 or 3 or 4 or 5 or 6

8 "variable*".tw.

9 "predict*".tw.

10 "determin*".tw.

11 "associat*".tw.

12 "factor*".tw.

13 8 or 9 or 10 or 11 or 12

14 7 and 13

15 systematic.tw.

16 14 and 15

17 limit 16 to yr="2000 -Current"

18 limit 17 to "review"

19 limit 18 to (human and english language)

C. **Search strategy used in** CINAHL

S1 MM patient compliance

S2 MM patient dropouts

S3 MM treatment refusal

S4 MM directly observed therapy

S5 TI adherence or AB adherence

S6 S1 and S5

S7 TI concordance or AB concordance

S8 S1 and S7

S9 S1 or S2 or S3 or S4 or S6 or S8

S10 TX factor*

S11 TX variable*

S12 TX predict*

S13 TX determin*

S14 TX associat*

S15 S10 or S11 or S12 or S13 or S14

S16 S9 and S15

S17 TX systematic

S18 S16 and S17

S19 S16 limit Syst Rev

S19 S16 or S19 limit English language

D. Search strategy used in Cochrane Library

#1 MeSH descriptor Patient Compliance explode all trees

#2 MeSH descriptor Patient Dropouts explode all trees

#3 MeSH descriptor Treatment Refusal explode all trees

#4 MeSH descriptor Directly Observed Therapy explode all trees
#5 MeSH descriptor Medication Adherence explode all trees
#6 (CONCORDANCE):ti,ab
#7 (#6 AND #1)
#8 (#1 OR #2 OR #3 OR #4 OR #5 OR #7)
#9 (variable OR variables):ti,ab,kw
#10 (predictor OR predictors):ti,ab,kw
#11 (determinant OR determinants):ti,ab,kw
#12 (association OR associations):ti,ab,kw
#13 (factor OR factors):ti,ab,kw
#14 (#9 OR #10 OR #11 OR #12 OR #13)
#15 (#8 AND #14)
#16 systematic:ti,ab,kw
#17 (#15 AND #16)
#18 (#17), from 2000 to 2009
#19 (variable*):ti,ab,kw
#20 (predictor*):ti,ab,kw
#21 (determinant*):ti,ab,kw
#22 (association*):ti,ab,kw
#23 (factor*):ti,ab,kw
#24 (#19 OR #20 OR #21 OR #22 OR #23)
#25 (#8 AND #24)
#26 (#25 AND #16)
#27 (#26), from 2000 to 2009
#28 (#18 OR #27)

E. Search strategy used in IPA

1 patient compliance.mp. [mp=title, subject heading word, registry word, abstract, trade name/generic name]
2 patient dropouts.mp. [mp=title, subject heading word, registry word, abstract, trade name/generic name]
3 treatment refusal.mp. [mp=title, subject heading word, registry word, abstract, trade name/generic name]
4 directly observed therapy.mp. [mp=title, subject heading word, registry word, abstract, trade name/generic name]
5 medication adherence.mp. [mp=title, subject heading word, registry word, abstract, trade name/generic name]
6 concordance.mp. [mp=title, subject heading word, registry word, abstract, trade name/generic name]

7 1 or 2 or 3 or 4 or 5 or 6

8 variable*.mp. [mp=title, subject heading word, registry word, abstract, trade name/generic name]

9 predict*.mp. [mp=title, subject heading word, registry word, abstract, trade name/generic name]

10 determin*.mp. [mp=title, subject heading word, registry word, abstract, trade name/generic name]

11 associat*.mp. [mp=title, subject heading word, registry word, abstract, trade name/generic name]

12 factor*.mp. [mp=title, subject heading word, registry word, abstract, trade name/generic name]

13 8 or 9 or 10 or 11 or 12

14 7 and 13

15 systematic.mp. [mp=title, subject heading word, registry word, abstract, trade name/generic name]

16 14 and 15

17 limit 16 to (english language and human and yr="2000 -Current")

F. **Search strategy used in** PsycINFO

medication adherence [KW] OR

directly observed therapy [KW] OR

treatment refusal [KW] OR

patient dropouts [KW] OR

patient compliance [KW] OR

concordance [KW] OR

(CONCORDANCE [TI] OR concordance[AB]) AND PATIENT COMPLIANCE [KW])

(no further limitations were used as this search strategy returned only 20 items)

Appendix 2:

Reviews characteristics and results

References	Field	Patient group	Databases searched	Years covered	Review design	Adherence definition	Adherence component	Treatment duration	Positive effect on adherence					Negative effect on adherence				
									Patient-related factors	Condition-related factors	Therapy-related factors	Socio/economic-related factors	HCT-related factors	Patient-related factors	Condition-related factors	Therapy-related factors	Socio/economic-related factors	HCT-related factors
**Bao et al 2009[3]**	opioid dependance	NS	CMKI, EMBASE, MEDLINE	1950-2007	MA	% of patients continuing treatment	P	long-term			+							
**Bramlage et al 2009[4]**	hypertension	NS	PubMed	1995 - 2008	SR	taking medication as prescribed, on time and at the correct dose	P	long-term			+							
**Brandes et al 2009[5]**	multiple sclerosis	NS	CINAHL, IPA, MEDLINE	1970 - 2008	SR	NS	NS	long-term	+	+				+		+		
**Broekmans et al 2009[6]**	non-malignant chronic pain	adults	Cochrane, CINAHL, MEDLINE, PsycINF	start - 2006	SR	taking medication corresponding the agreed recommendations of the health care provider	I	long-term						+	+	+		+
**Charach et al 2008[7]**	ADHD	children	CINAHL, EMBASE, MEDLINE, PsycINFO	1950 – 2008	SR	consistent long-term use of medication; while non-adherence can be either consistent nonuse	P	long-term	+	+	+		+	+		+	+	+

References	Field	Patient group	Databases searched	Years covered	Review design	Adherence definition	Adherence component	Treatment duration	Positive effect on adherence					Negative effect on adherence				
									Patient-related factors	Condition-related factors	Therapy-related factors	Socio/economic-related factors	HCT-related factors	Patient-related factors	Condition-related factors	Therapy-related factors	Socio/economic-related factors	HCT-related factors
						once discontinued or inconsistent use with periods of stopping and starting												
**Chia et al 2006**[8]	miscellaneous diseases	elderly	Academic Search Primer, Adis, Anthropology Plus, Cumulative Index to Allied Health Literature, Ethnic Newswatch, IPA, EMBASE, MEDLINE, PsycINFO, Sociological Abstracts	1975 - 2005	SR	NS	I	long-term	+	+	+		+	+				
**Claxton et**	miscellaneous	NS	Cochrane, Health &	1986 - 2000	SR	taking medication	I	long-term								+		

References	Field	Patient group	Databases searched	Years covered	Review design	Adherence definition	Adherence component	Treatment duration	Positive effect on adherence					Negative effect on adherence				
									Patient-related factors	Condition-related factors	Therapy-related factors	Socio/economic-related factors	HCT-related factors	Patient-related factors	Condition-related factors	Therapy-related factors	Socio/economic-related factors	HCT-related factors
**al 2001**[9]	diseases		Psychosocial Instruments, HealthStar, MEDLINE, PsycINFO			as prescribed; partial adherence - taking less than the prescribed amount of medication or taking the medication at inappropriate intervals; total non-adherence - discontinuation of treatment.												
**Connor et al 2004** [10]	miscellaneous diseases	adults	CINAHL, Cochrane, EMBASE, IPA, MEDLINE, metaRegister of Controlled Trials	1966 - 2003	SR	NS	I	long-term			+							
**Costello et al 2008**[11]	multiple sclerosis	NS	MEDLINE	NS	SR	comprises 3 distinct subcategories:	I	long-term	+		+	+		+	+	+	+	+

References	Field	Patient group	Databases searched	Years covered	Review design	Adherence definition	Adherence component	Treatment duration	Positive effect on adherence					Negative effect on adherence				
									Patient-related factors	Condition-related factors	Therapy-related factors	Socio/economic-related factors	HCT-related factors	Patient-related factors	Condition-related factors	Therapy-related factors	Socio/economic-related factors	HCT-related factors
						acceptance, persistence, and compliance												
**Cramer et al 2004[12]**	diabetes	NS	Cochrane, Current Contents, Health & Psychosocial Instruments, MEDLINE	1966 - 2003	SR	taking medication as prescribed and/or agreed between the patients and the health care provider. various operational definitions	I	long-term	+	+					+			
**DiMatteo et al 2007[13]**	miscellaneous diseases	children, adults	old MEDLINE, PsychLit, PubMed	1948–2005	MA	various operational definitions	I + P	long-term	+				+					
**DiMatteo et al 2000[14]**	miscellaneous diseases	children, adults	MEDLINE, PsychLit	1968-1998	MA	various operational definitions	I	long-term						+				
**DiMatteo et al 2004[15]**	miscellaneous diseases	children, adults	MEDLINE, PsychLit	1948 – 2001	MA	various operational definitions	I	long-term				+					+	
**DiMatteo et al**	miscellaneous	children, adults	Index Medicus,	1948 – 1998	MA	various operational	I	long-term	+	+					+			

References	Field	Patient group	Databases searched	Years covered	Review design	Adherence definition	Adherence component	Treatment duration	Positive effect on adherence					Negative effect on adherence				
									Patient-related factors	Condition-related factors	Therapy-related factors	Socio/economic-related factors	HCT-related factors	Patient-related factors	Condition-related factors	Therapy-related factors	Socio/economic-related factors	HCT-related factors
**2004[16]**	diseases		MEDLINE, PsychLit			definitions												
**Fogarty et al 2002[17]**	HIV	NS	Cochrane, PsycINFO, MEDLINE, Sociofile	start - 1999	SR	various operational definitions	P	long-term	+				+	+		+	+	
**Gold et al 2006[18]**	osteoporosis	NS	MEDLINE	1975 - 2005	SR	various operational definitions	P	long-term	+				+		+	+	+	
**Gonzalez et al 2008[19]**	diabetes	children, adults	MEDLINE, PsycINFO	1950 – 2008	MA	various operational definitions	NS	long-term						+				
**Hirsch-Moverman et al 2008[20]**	tuberculosis	adults	MEDLINE, PsycINFO, PubMed	1997 - 2007	SR	NS	I	long-term	+	+	+	+		+	+			
**Hodari et al 2006[21]**	dermatological diseases	NS	PubMed	1985 - 2005	SR	various operational definitions	I	long-term	+					+	+	+		+
**Iskedjian et al 2002[22]**	hypertension	adults	EMBASE, MEDLINE, IPA	1980 – 1998	MA	proportion of patients who had taken >80% of doses	I	long-term			+							

References	Field	Patient group	Databases searched	Years covered	Review design	Adherence definition	Adherence component	Treatment duration	Positive effect on adherence					Negative effect on adherence				
									Patient-related factors	Condition-related factors	Therapy-related factors	Socio/economic-related factors	HCT-related factors	Patient-related factors	Condition-related factors	Therapy-related factors	Socio/economic-related factors	HCT-related factors
**Jacobsen et al 2009[23]**	cancer	adults	Cochrane, EMBASE, MEDLINE, Web of Science	start - 2006	SR	NS	I	long-term					+	+				
**Jindal et al 2003[24]**	post kidney transplant patients	NS	MEDLINE	start - 2001	SR	NS	I	long-term	+					+	+			
**Julius et al 2009[25]**	psychiatric disorders	NS	Ovid, Medline	start - 2008	SR	extent to which a patient's behavior coincides with medical or prescribed health advice	I	long-term	+			+	+	+		+	+	+
**Kahana et al 2008[26]**	post transplant patients	children	Psyc-INFO, PUBMED/ MEDLINE	NS	SR	various operational definitions	I	long-term						+		+	+	
**Karamanidou et al 2008[27]**	end stage renal disease	adults	CINAHL, EMBASE, PsycINFO, MEDLINE	1950 - 2006	SR	NS	I	long-term	+			+		+			+	
**Kruk et al**	miscellaneous	NS	IPA, MEDLINE	2000 - 2005	SR	various operational	I	long-term			+							

References	Field	Patient group	Databases searched	Years covered	Review design	Adherence definition	Adherence component	Treatment duration	Positive effect on adherence					Negative effect on adherence				
									Patient-related factors	Condition-related factors	Therapy-related factors	Socio/economic-related factors	HCT-related factors	Patient-related factors	Condition-related factors	Therapy-related factors	Socio/economic-related factors	HCT-related factors
**2006[28]**	diseases					definitions												
**Lacro et al 2002[29]**	schizofrenia	NS	HealthSTAR, MEDLINE, PsycINFO	1980 – 2002#	SR	taking medications as prescribed at least 75% of the time	I	long-term						+	+	+		+
**Lanouette et al 2009[30]**	psychiatric disorders	adults (US Latinos)	M EDLINE, PsycINFO	1980 – 2009#	SR	various operational definitions	I + P	long-term	+	+	+	+		+				+
**Lee et al 2006[31]**	diabetes	NS	MEDLINE, Pubmed	1990 -2005	SR	various operational definitions	I + P	long-term			+						+	
**Lewiecki et al 2007[32]**	osteoporosis	adults	Cochrane, MEDLINE	1987 - 2007	SR	NS	I	long-term		+				+		+		
**Lovejoy et al 2009[33]**	HIV	HIV-positive adults	MEDLINE, PsycINFO	Start - 2008	SR	various operational definitions	I	long-term						+				
**Malta et al 2008[34]**	HIV	HIV-positive drug users	AIDSLINE, AMED, CINAHL, Cochrane, MEDLINE	1996 - 2007	SR	various operational definitions	I + P	long-term					+	+		+		

References	Field	Patient group	Databases searched	Years covered	Review design	Adherence definition	Adherence component	Treatment duration	Positive effect on adherence					Negative effect on adherence				
									Patient-related factors	Condition-related factors	Therapy-related factors	Socio/economic-related factors	HCT-related factors	Patient-related factors	Condition-related factors	Therapy-related factors	Socio/economic-related factors	HCT-related factors
			, TOXNET, Web of Science															
**Mills et al 2006[35]**	HIV	NS	AMED, Campbell Collaboration Cochrane, CINAHL, EMBASE, ERIC, MEDLINE, NHS EED	Start - 2005	SR	NS	NS	long-term	+		+	+	+	+		+	+	+
**Munro et al 2007[36]**	tuberculosis	NS	Academic Search Premier, CINAHL, EMBASE, ERIC, MEDLINE, Pre-CINAHL, PapersFirst, PsycINFO, PubMed, dissertatio	1966 - 2005	SR	NS	I + P	long-term	+	+	+	+	+	+	+	+	+	+

References	Field	Patient group	Databases searched	Years covered	Review design	Adherence definition	Adherence component	Treatment duration	Positive effect on adherence					Negative effect on adherence				
									Patient-related factors	Condition-related factors	Therapy-related factors	Socio/economic-related factors	HCT-related factors	Patient-related factors	Condition-related factors	Therapy-related factors	Socio/economic-related factors	HCT-related factors
			n abstracts, sociological abstracts, social services abstracts, PAIS international, Health Source: Nursing/Academic, ScienceDirect, Social Science full text, Social science citation expanded, social science citation index, arts and															

References	Field	Patient group	Databases searched	Years covered	Review design	Adherence definition	Adherence component	Treatment duration	Positive effect on adherence					Negative effect on adherence				
									Patient-related factors	Condition-related factors	Therapy-related factors	Socio/economic-related factors	HCT-related factors	Patient-related factors	Condition-related factors	Therapy-related factors	Socio/economic-related factors	HCT-related factors
			humanities citation index															
**Nosé et al 2003[37]**	psychosis	NS	MEDLINE, PsycINFO	1980 – 2003#	SR	the extent to which a person's behaviour coincides with the medical advice given	I	long-term	+	+		+	+	+	+	+	+	
**Oehl et al 2000[38]**	psychosis	NS	MEDLINE	NS	SR	NS	NS	long-term	+		+	+		+	+	+	+	+
**Olthoff et al 2005[39]**	glaucoma	NS	CINAHL, Cochrane MEDLINE, EMBASE, PsycINFO	start - 2004	SR	the degree of correspondence between the prescribed regimen and a patient's actual dosing history	I + P	long-term	+	+			+	+		+		+
**Pampallona et al 2002[40]**	depression	NS	Cochrane, Current Contents, MEDLINE, PsycInfo	1973 - 1999	SR	the extent to which a person's behavior conforms to medical or health advice	I	long-term						+	+	+		+
**Parienti et**	HIV	NS	PubMed, recent	NS	MA	mean adherence	I	long-term			+							

References	Field	Patient group	Databases searched	Years covered	Review design	Adherence definition	Adherence component	Treatment duration	Positive effect on adherence					Negative effect on adherence				
									Patient-related factors	Condition-related factors	Therapy-related factors	Socio/economic-related factors	HCT-related factors	Patient-related factors	Condition-related factors	Therapy-related factors	Socio/economic-related factors	HCT-related factors
**al 2009**[41]			HIV science conferences			rate, defined as: (total number of doses taken/ total number of doses prescribed)× 100												
**Ramos et al 2009**[42]	HIV	children	MEDLINE, EMBASE, conference abstract	NS	SR	NS	NS	long-term			+							
**Reisner et al 2009**[43]	HIV	youth (13-24 years)	PubMed, PsycINFO, MEDLINE	1999 - 2008	SR	various operational definitions	I + P	long-term	+	+	+	+	+	+	+	+	+	
**Ruddy et al 2009**[44]	cancer	children	PubMed	NS	SR	the degree or extent of conformity to the recommendations about day-to-day treatment by the provider with respect to the timing, dosage, and frequency	I + P	long-term	+	+			+	+	+	+	+	
**Santarlasci et al**	schizophrenia	NS	MEDLINE	1966 - 2002	SR	a dropout or treatment discontinuation	P	long-term								+		

References	Field	Patient group	Databases searched	Years covered	Review design	Adherence definition	Adherence component	Treatment duration	Positive effect on adherence					Negative effect on adherence				
									Patient-related factors	Condition-related factors	Therapy-related factors	Socio/economic-related factors	HCT-related factors	Patient-related factors	Condition-related factors	Therapy-related factors	Socio/economic-related factors	HCT-related factors
**2003[45]**						was used instead of adherence												
**Schmid et al 2009[46]**	end stage renal disease	adults	PubMed, Medline	1971-2008	SR	various operational definitions	NS	long-term	+		+			+	+		+	+
**Van Der Wal et al 2005[47]**	heart failure	adults	CINAHL, MEDLINE	1988 - 2003	SR	extent to which a persons' behaviour (in terms of taking medication, following diet or executing life style changes) coincides with the 'clinical prescription'	I	long-term	+	+	+		+			+		
**Vermeire E et al 2001[48]**	miscellaneous diseases	NS	EMBASE, ERIC, PsycINFO, MEDLINE, Sociological abstracts, Dissertation	1975 - 1999	SR	various operational definitions	I	long-term	+	+		+	+	+	+	+		+

References	Field	Patient group	Databases searched	Years covered	Review design	Adherence definition	Adherence component	Treatment duration	Positive effect on adherence					Negative effect on adherence				
									Patient-related factors	Condition-related factors	Therapy-related factors	Socio/economic-related factors	HCT-related factors	Patient-related factors	Condition-related factors	Therapy-related factors	Socio/economic-related factors	HCT-related factors
			abstracts															
**Vik et al 2004[49]**	miscellaneous diseases	elderly	IPA, MEDLINE, PubMed,	1966 - 2002	SR	the extent to which a person's behavior (in terms of taking medication, following diets, or executing lifestyle changes) coincides with medical or health advice	I	long-term					+	+		+		+
**Vreeman et al 2008[50]**	HIV	children	EMBASE, MEDLINE, relevant websites	1966 - 2007	SR	various operational definitions	I	long-term	+					+		+	+	
**Weiner et al 2008[51]**	cystic fibrosis	children, adults	MEDLINE, selected conference abstracts	1990 - 2007	SR	The extent to which a patient's behavior coincides with medical advice	I	long-term	+		+		+	+		+	+	
**Wetzels et al 2004[52]**	hypertension	NS	MEDLINE, PubMed, EMBASE	1985 - 2003	SR	taking compliance - % of prescribed doses taken: (total number of recorded	I	long-term			+					+		

References	Field	Patient group	Databases searched	Years covered	Review design	Adherence definition	Adherence component	Treatment duration	Positive effect on adherence					Negative effect on adherence				
									Patient-related factors	Condition-related factors	Therapy-related factors	Socio/economic-related factors	HCT-related factors	Patient-related factors	Condition-related factors	Therapy-related factors	Socio/economic-related factors	HCT-related factors
						medication events/total number of prescribed doses) X 100%; correct dosing - % of days on which the correct number of doses was taken: (the total number of days with recorded medication events as prescribed/total number of monitored days) X 100%.												
**Yeung et al 2005[53]**	malaria	children, adults	EMBASE, PubMed, web sites	NS	SR	full adherence - the medication was taken at a dose and duration that was considered to be within a range that would result in the same efficacy as	I	long-term			+							

References	Field	Patient group	Databases searched	Years covered	Review design	Adherence definition	Adherence component	Treatment duration	Positive effect on adherence					Negative effect on adherence				
									Patient-related factors	Condition-related factors	Therapy-related factors	Socio/economic-related factors	HCT-related factors	Patient-related factors	Condition-related factors	Therapy-related factors	Socio/economic-related factors	HCT-related factors
						the recommended regime; also various operational definitions												

Abbreviations: I - implementation, P - persistence, MA – meta-analysis, NS - not stated, SR – systematic review
# - search end year not provided explicitly, the publication year was given, instead
